# Faecal transplantation for the treatment of *Clostridium difficile* infection in a marmoset

**DOI:** 10.1186/s12917-017-1070-z

**Published:** 2017-05-31

**Authors:** Yumiko Yamazaki, Shinpei Kawarai, Hidetoshi Morita, Takefumi Kikusui, Atsushi Iriki

**Affiliations:** 1 Advanced Research Centres, Keio University, 35 Shinanomachi, Shinjuku-ku, Tokyo, 160-8582 Japan; 2 Laboratory for Symbolic Cognitive Development, RIKEN BSI, 2-1 Hirosawa, Wako-shi, Saitama, 351-0198 Japan; 3 Laboratory of Small Animal Clinics, Veterinary Teaching Hospital, Azabu University, 1-17-71 Fuchinobe, Chuo-ku, Sagamihara-shi, Kanagawa 252-5201 Japan; 4 Graduate School of Environmental and Life Science, Okayama University, 1-1-1 Tsushimanaka, Kita-ku, Okayama-shi, Okayama, 700-8530 Japan; 5 Companion Animal Research, School of Veterinary Medicine, Azabu University, 1-17-71 Fuchinobe, Chuo-ku, Sagamihara-shi, Kanagawa 252-5201 Japan; 6 RIKEN-NTU Research Centre for Human Biology, Nanyang Technological University, Singapore, 639798 Singapore

**Keywords:** *Clostridium difficile*, Common marmoset, Diarrhoea, Faecal microbiota transplantation, Metronidazole

## Abstract

**Background:**

The common marmoset has been used as an experimental animal for various purposes. Because its average weight ranges from 250 to 500 g, weight loss quickly becomes critical for sick animals. Therefore, effective and non-stressful treatment for chronic diseases, including diarrhoea, is essential.

**Case presentation:**

We report a case in which faecal microbiota transplantation (FMT) led to immediate recovery from chronic and recurrent diarrhoea caused by *Clostridium difficile* infection. A male common marmoset experienced chronic diarrhoea after antibiotic treatments. The animal experienced severe weight loss, and a faecal sample was confirmed to be *C. difficile*-positive but was negative for protozoa. Metronidazole was partially effective at the first administration but not after the recurrence of the clinical signs. Then, oral FMT was administered to the subject by feeding fresh faeces from healthy individuals mixed with the marmoset’s usual food. We monitored the faeces by categorization into four groups: normal, loose, diarrhoea, and watery. After the first day of FMT treatment, the marmoset underwent a remarkable recovery from diarrhoea, and after the fourth day of treatment, a test for *C. difficile* was negative. The clinical signs did not recur. The marmoset recovered from sinusitis and bilateral dacryocystitis, which also did not recur, as a by-product of the improvement in its general health caused by the cessation of diarrhoea after the FMT.

**Conclusion:**

This is the first reported case of successful treatment of a marmoset using oral FMT. As seen in human patients, FMT was effective for the treatment of recurrent *C. difficile* infection in a captive marmoset.

## Background

*Clostridium difficile* is a major cause of severe diarrhoea, especially in hospitalized patients receiving antibiotic therapy [1]. Metronidazole is the first choice for *C. difficile* infections [1] in humans and small animals [2]. However, some patients suffer from recurrent infections after repeated treatments [1, 3].

Faecal microbiota transplantation (FMT) is an emergent treatment for diarrhoea caused by various pathogens [4]. Although the success rate of the therapy is variable and the techniques applied in the studies vary widely [5], a few studies have reported immediate recovery after several recurrent *C. difficile* infections (e.g., within 24 h after FMT) [6].

Here, we present a report of successful recovery from severe chronic diarrhoea after FMT in a male common marmoset (*Callithrix jacchus*). This species of small New World monkey has been used as an experimental animal for various purposes, including medical [7], neuroscientific [8], and cognitive studies [9], and the recent establishment of genetically modified animals [10] has enhanced the use of this species in additional research areas. Because their average weight ranges from 250 to 500 g, weight loss quickly becomes critical for sick animals. Therefore, effective and non-stressful treatment for chronic diseases, such as diarrhoea, is necessary. Thus, we administered oral FMT to an animal with chronic diarrhoea caused by *C. difficile*; this is a novel method for the treatment of gut microbiota disturbances in this species.

## Case presentation

**Fig. 1:**
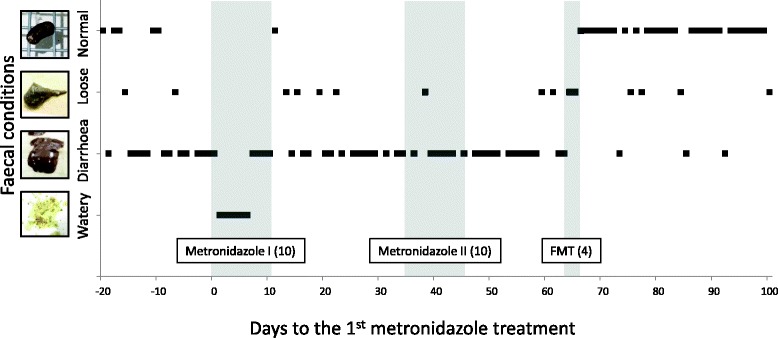
Time course of diarrhoea before, during, and after the treatments. The “0” on the *horizontal axis* indicates the first day of the first metronidazole treatment. The faeces were divided into four categories (normal, loose, diarrhoea, and watery) as represented in the pictures next to the *vertical axis*. *Shaded vertical lines* show the two periods of metronidazole treatment and the faecal microbiota transplantation. The *numbers* next to the names of the treatments indicate the period in days

A male colony-bred common marmoset weighing 420 g that was 2 y and 2 m of age experienced chronic diarrhoea after a 6-day treatment with ampicillin (Viccillin S-100, Meiji Seika Pharma, Tokyo, Japan, 25 mg/h, i.m., every 24 h) to prevent infection after craniotomy for neural recording; this study was a component of the neurocognitive study projects approved by the Animal Experiment Committees at the RIKEN Brain Science Institute and was conducted in accordance with the Guidelines for Conducting Animal Experiments of the RIKEN Brain Science Institute (H27–2-303). This animal had been housed singly for a few months in preparation for the surgery. The breeding room was maintained at 26–28 °C with 50–60% humidity. Due to the chronic diarrhoea, the animal experienced severe weight loss (BW 360 g; approximately 60 g lost compared to the pre-surgery weight) and was given an oral nutritional supplement (ELENTAL P, EA Pharma, Tokyo, Japan, 2 g per day) and a *Lactobacillus* preparation (Bioymbuster, Kyoritsu Seiyaku, Tokyo, Japan﻿, 50mg/h, p.o., per d﻿ay) in addition to the standard food regimen. The subject recovered from diarrhoea 1 month after termination of the antibiotic. This antibiotic has also been shown to cause *C. difficile*-associated disease in human patients [11]. A faecal sample collected 3 weeks after surgery was confirmed to be *C. difficile*-positive by anaerobic culture. The sample was negative for protozoa by zinc sulphate flotation performed at a commercial veterinary diagnostic laboratory (Monoris Inc., Tokyo, Japan). Diarrhoea was intermittent (occurrence on 11.3% of days, no watery appearance) for one and a half months after treatment. However, after that period, diarrhoea recurred on 65% of 20 days and was often watery and muddy (dark brown appearance with little solid content). At this point, we tested the faecal sample (Techlab C Diff Quick Chek Complete, Alere, Chiba, Japan) and confirmed that it was positive for *C. difficile* antigen and toxin. Figure 1 shows the time course of the disease. We categorized the faeces into four groups as follows: normal (solid, with little liquid), loose (blobby with liquid but still formed), diarrhoea (mostly blobby, large amount of liquid, partially muddy), and watery (almost liquid, with small pieces of solids), as photographically represented next to the y-axis in Fig. 1. When there were no faeces, no score was assigned. We scored each day by the worst stool of the day; for instance, if both normal faeces and diarrhoea occurred during a day, then we recorded “diarrhoea” for that day.

**Fig. 2:**
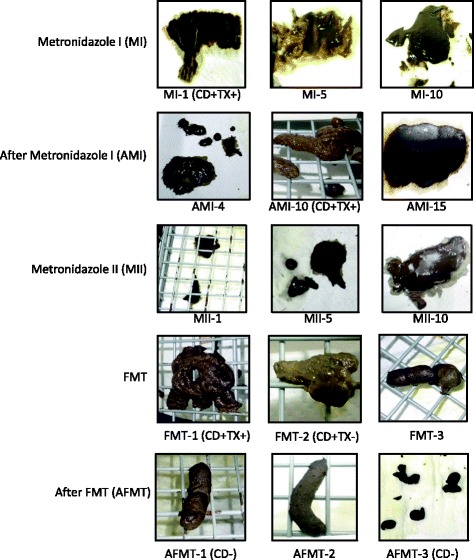
Representative faecal samples during and after the treatments. Under each picture, the *numbers* next to the abbreviated names of the treatments (e.g., MI) show the day within a given treatment or phase. The results of the tests for *Clostridium difficile* antigen and toxin are shown in *parentheses* next to the legend. CD: *C. difficile* antigen; TX: *C. difficile* toxin

The marmoset received metronidazole (Flagyl 250 mg, Shionogi, Osaka, Japan, 25 mg/h, every 24 h) [12] for 10 days (2 5-day periods separated by 2 weekend days). The diarrhoea decreased slightly during and after the medication, as illustrated by “After Metronidazole I (AMI) -10” in Fig. 2, although the *C. difficile* test was still positive for antigen and toxin. However, the diarrhoea quickly worsened within 10 days after termination of the medication. We re-administered the metronidazole 23 days after the end of the first medication cycle for 10 days as described above, with little evidence of recovery (Figs. 1 and 2).

Because the metronidazole was no longer effective, we decided to perform a FMT. Faeces from healthy individuals were administered orally by mixing with the pellet food (CMS-1M, CLEA Japan, Tokyo, Japan). Specifically, 3 g of faeces from marmosets, 4 g of powdered marmoset food, 2 g of honey, and 8 ml of lukewarm water were mixed to make a wet mash. The donors were seven healthy marmosets with no history of medication for at least 3 months. Multiple donors were selected to increase the diversity of the microbiota in the faeces, which is thought to be key for effective FMT [4]. Faecal samples from the marmosets were collected within 30 min after defecation. The FMT treatment continued for four consecutive days, and at least two donors were selected per day. This method was chosen because the faecal samples were not consistently available.

The consistency of the subject’s faeces changed quickly (Fig. 1). The faeces became more solid the day after the first FMT treatment (FMT-1 in Fig. 2), although the tests were still positive for *C. difficile* antigen and toxin. After the second treatment, the tests were *C. difficile*-positive but toxin-negative. The faeces were almost normal after the third treatment (FMT-3 in Fig. 2). Finally, the test results were negative for both *C. difficile* antigen and toxin after the fourth day of FMT treatment (After FMT (AFMT)-1 in Fig. 2). Thereafter, the marmoset completely recovered from chronic diarrhoea and remained healthy for the subsequent 10 months, as shown in Fig. 1. No adverse effects were observed during or after the FMT treatment. *C. difficile* infection has not been observed in other animals in that breeding room.

**Fig. 3:**
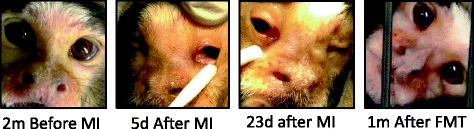
Nasal and ocular signs before and after the treatments. *Swelling* between the eyes and nose was prominent after the first administration of metronidazole; FMT: faecal microbiota transplantation; m: month; d: day

In addition to the cessation of diarrhoea after the FMT treatment, the marmoset recovered from sinusitis and bilateral dacryocystitis, which had developed after the surgery for unknown reasons. The presence of sinusitis, epiphora, and mild conjunctivitis and the location of the swelling at the inner corners of eyes (especially the areas around the lacrimal punctum) indicated a tentative diagnosis of dacryocystitis. A bacteriological examination (Monoris Inc., Tokyo, Japan) of the discharge from the nose 1 month after the surgery confirmed the presence of a Gram-negative bacillus that showed sensitivity to enrofloxacin. Therefore, enrofloxacin (Baytril 15 mg, Bayer, Tokyo, Japan, 2.5 mg/kg, p.o., every 24 h) was administered for 10 days. Three months after this treatment, which was concurrent with the recovery from diarrhoea, the clinical signs became worse, and the swelling around the eyes and nose was significantly worse than the signs observed 2 months prior to the treatment (Fig. 3). The swelling did not improve after the first or second metronidazole treatment. On the first day of FMT, a large amount of nasal mucus was evident. However, the marmoset rapidly recovered, with no nasal mucus or lacrimation by the third day of FMT. Finally, these clinical signs resolved 1 month after treatment (Fig. 3), with no recurrence during the 10 months after FMT.

## Discussion and Conclusions

Chronic and severe diarrhoea caused by *C. difficile* infection was terminated after 4 days of oral intake of faecal microbiota collected from healthy individuals. The subject readily accepted the treatment because the faeces were mixed with the usual marmoset food. This treatment showed marked benefits without the stress of drug administration by intragastric tube or a probe. Additionally, the FMT food was more palatable than metronidazole (which is extremely bitter); thus, a full dose could be administered every day (we determined that the food was palatable because the subject continued to eat the FMT food; if the food was not palatable, like metronidazole, the subject stopped eating, started salivating excessively, and wiped the food on the cage wires or perches). This immediate recovery was similar to the recovery observed in some human subjects [4, 6], with improvement starting within 24 h after the first treatment. These improvements in the clinical signs were correlated with the recovery from sinusitis and dacryocystitis. However, because no direct causal relationship existed between these clinical signs and the FMT, the general improvement in the animal’s health condition after the cessation of diarrhoea most likely contributed to its recovery from these signs.

Of course, many questions remain unanswered. For example, we do not know the origin of the *C. difficile* infection. To this end, an analysis of the microbiota of the infected and healthy animals is needed. A comparison of the microbiota of the donors with the microbiota of the patient after recovery could reveal the most desirable distribution of microbiota for FMT. Information on the Bifidobacteria in the common marmoset microbiome has been accumulating [13–16]. Bifidobacteria are widely used as probiotic organisms and may confer a health benefit to the host when an effective amount is administered to balance the host microbiome. This information would support a more detailed analysis of the healthy microbiome of this animal.

In conclusion, FMT appeared to be effective for the treatment of recurrent *C. difficile* infection in a captive marmoset, as seen in human patients. This small primate could be a valid model for the study of *C. difficile* infections and effective treatments, including FMT, in human patients.

## Abbreviations

FMT: Faecal microbiota transplantation
*C. difficile*: *Clostridium difficile*
